# Conventional and genetic associations between resting heart rate, cardiac morphology and function as assessed by magnetic resonance imaging: Insights from the UK biobank population study

**DOI:** 10.3389/fcvm.2023.1110231

**Published:** 2023-03-17

**Authors:** Yao Ma, Mengyao Qi, Kexin Li, Yuan Wang, Fuxian Ren, Dengfeng Gao

**Affiliations:** ^1^ Cardiology Diseases Department, Xi’an Jiaotong University Second Affiliated Hospital, Xi’an, China; ^2^ Department of Cardiology, Meishan Brach of the Third Affiliated Hospital, Yanan University School of Medical, Meishan, China

**Keywords:** resting heart rate, cardiac magnetic resonance, left ventricular remodeling, heart failure, mendelian randomization

## Abstract

**Aim:**

To examine the direction, strength and causality of the associations of resting heart rate (RHR) with cardiac morphology and function in 20,062 UK Biobank participants.

**Methods and results:**

Participants underwent cardiac magnetic resonance (CMR) and we extracted CMR biventricular structural and functional metrics using automated pipelines. Multivariate linear regression adjusted for the main cardiovascular risk factors and Two-sample Mendelian Randomization analyses were performed to assess the potential relationship, grouped by heart rate and stratified by sex. Each 10 beats per minute increase in RHR was linked with smaller ventricular structure (lower biventricular end-diastolic volume and end-systolic volume), poorer left ventricular (LV) function (lower LV ejection fraction, global longitude strain and global function index) and unhealthy pattern of LV remodeling (higher values of myocardial contraction fraction), but there is no statistical difference in LV wall thickness. These trends are more pronounced among males and consistent with the causal effect direction of genetic variants interpretation. These observations reflect that RHR has an independent and broad impact on LV remodeling, however, genetically-predicted RHR is not statistically related to heart failure.

**Conclusion:**

We demonstrate higher RHR cause smaller ventricular chamber volume, poorer systolic function and unhealthy cardiac remodeling pattern. Our findings provide effective evidence for the potential mechanism of cardiac remodeling and help to explore the potential scope or benefit of intervention.

## 1. Introduction

Epidemiological studies consistently show that resting heart rate (RHR) is a predictor of cardiovascular morbidity, mortality, and all-cause mortality in a wide range of subjects, as well as a controllable therapeutic target to improve the prognosis of heart failure (1–4). Left ventricular (LV) remodeling is a clinical characteristic in which morphological changes in the LV development and progression resulting in ventricular dysfunction (5). Elevated RHR is associated with all steps of the continuum of adverse cardiovascular events, including worsening cardiometabolic risk, target organ damage, accelerated arteriosclerosis, and decreased ejection fraction (3, 6).

However, whether inappropriate RHR is a causative factor for changes in ventricular structure has rarely been studied. Although different patterns of cardiovascular disease (CVD) related to sex are widely recognized, the association of RHR-mediated sex differences is unclear (7).

Recently, cardiac magnetic resonance (CMR) has been recognized as a better standard compared to echocardiography for assessing heart morphology and function because it can provide more accurate and detailed measurements, particularly in patients with poor RV assessment and image quality (8–10). Mendelian randomization (MR), as an extension to cross-sectional research, is a method that can evidence the causal effect of modifiable lifestyle or environmental factors on complicated disease phenotypes predicted by a set of genetic instruments (11). MR analysis is less susceptible to be influenced by confounding factors and reverse causation because genetic variants are fixed at the time of conception and segregate randomly from parent to offspring.

Therefore, we sought to demonstrate the association in the UK Biobank population study stratified by sex between RHR and biventricular morphology derived from CMR imaging and assess the potential causal relevance of RHR on cardiac remodeling using MR techniques, aiming to provide ideas for the mechanism of RHR affecting the pathogenesis of CVD and theoretical support for the treatment of heart rate control in CVD (12).

## 2. Methods

### 2.1. Study population

The UK Biobank (http://www.ukbiobank.ac.uk) is a large population-based study cohort that incorporated data from more than 500,000 middle-aged participants between 2006 and 2010 (13). Information was collected on characteristics such as sociodemographic, lifestyle, environmental factors, genotype, medical history, physical measurements, and CMR- derived cardiac phenotypes (12). All procedures were conducted in compliance with the ethical principles of medical research as set forth in the World Health Organization's Declaration of Helsinki, and all participants signed the appropriate informed consent. This study adhered to the STROBE (Strengthening the Reporting of Observational Studies in Epidemiology) guideline. The CMR imaging study initiated in 2014 was designed to scan a subset of 100,000 participants (14–16). The resulting massive imaging datasets was tested against published methods for cardiac image quantification.

### 2.2. Measurement of RHR and blood pressure

RHR measurements were performed according to standard operating procedures of the UK Biobank baseline assessment visits and described in detail in a dedicated document (17). Measurements were performed using an Omron 705 IT Sphygmomanometer (OMRON Healthcare Europe B.V.; Kruisweg 577 2132 NA Hoofddorp, Netherlands). Measurements for RHR and blood pressure were performed by specialized nurses in a dedicated room after 15 min of participant sitting still in a relaxed state from the left arm. The readings were generated by an automatic machine electronic recording. Secondary measurements in the repeated condition were performed among all individuals. The average of the two readings of RHR and blood pressure was used for analysis in this study.

### 2.3. CMR imaging analysis

The CMR protocol of UK Biobank has already been described (15, 16). All examinations were performed on a 1.5 Tesla scanner (MAGNETOM Aera, Syngo Platform VD13A, Siemens Healthcare, Erlangen, Germany). CMR metrics were obtaind from a fully automated image analysis pipeline that had previously developed and validated in a large-scale subset (14, 18). Details of the reproducibility performance of the automated algorithm can be found in a specialized publication (9). We included metrics from the first 26,892 UK Biobank CMR studies in the present analysis. The available metrics are as follows: LV and RV end-diastolic volume (EDV), end-systolic volume (ESV), ejection fraction (EF), stroke volume (SV), LV mass, LV wall thickness (WT) and global longitudinal strain (GLS). Past evidence suggested that GLS is more sensitive to LV dysfunction than LVEF and can provide additional prognostic information (19). LV global function index (LVGFI) was considered as a better indicator of cardiac function than LVEF for predicting heart failure and various cardiovascular disease events (20). LVGFI (%) was defined as LVSV/LV global volume × 100, where LV global volume was calculated as the sum of the LV mean chamber volume [(LVEDV + LVESV)/2] and myocardial volume (LV mass/density). LV Density was specified as 1.05 g/ml. We also considered myocardial contraction fraction (MCF) as a measure of myocardial shortening by calculating the ratio of stroke volume to myocardial volume (21). A lower MCF is more prone to pathological LV hypertrophy.

### 2.4. Selection of genetic variants

Genetic variants of RHR were selected from an authoritative published study from HRgene consortium with significantly genome-wide associations (*P* < 5 × 10^−8^) (22). In brief, a 2-phase meta-analysis of genome-wide association studies was performed with imputed genotype data of RHR in up to 85,787 mixed Europeans. Independent genetic variants were identified by clumping (linkage disequilibrium *r*^2^ < 0.01 within a ± 250-kb window). Coding alleles were defined as alleles associated with higher RHR. In general, thirty-two single nucleotide polymorphisms (SNPs) were identified to predict RHR, which involved in the genetics of embryonic heart development and the pathophysiology of a variety of heart diseases. The identified variants are shown in Supplementary Table S1 in detail.

### 2.5. Covariates

All UK Biobank participants completed a comprehensive baseline health and lifestyle assessment through self-report questionnaires, interviews and physical measurements. The following confounding factors were considered for the accuracy of RHR and population stratification. Questionnaire-based data included: sex, age, ethnicity, socioeconomic status (Townsend Deprivation Index), smoking and alcohol status (never, previous and current), and physical activity level. Measurements of body mass index (BMI) were derived from general body measurement data. We also considered a self-reported history of CVD (high blood pressure, angina, heart attack, or stroke), diabetes mellitus, and use of heart rate altering drugs (beta-blockers, oral nitrates, and variety of related medications) (Supplementary Table S2). Essential hypertension (I10) and heart failure (I50–I59) patients were defined according to International Statistical Classification of Diseases-10th Revision (ICD-10) codes of the National Inpatient Hospital Data Statistics.

### 2.6. Statistical analysis

#### 2.6.1. Clinical analyses

After all variables were examined for normal distribution, abnormal values exceeding four times the standard deviation of the mean were excluded. Patients with an RHR greater than 150 bpm were excluded by expertise and normality assessment. Univariate and multivariate linear regression models were constructed, and regression coefficients (β) with corresponding 95% confidence intervals (95% CI) were calculated for the association between RHR as the determinant and LV and RV parameters as outcome variables. The multivariable model was adjusted according to covariates mentioned above. In the interest of simplicity, GLS values in the analysis process are reported as absolute (i.e., positive) values. Sex interactions were included in the regression models to investigate whether RHR was more significantly correlated with cardiovascular morphology and function in men or women. For further study on the relationship between the RHR level and CMR parameters, participants were divided into four groups by RHR level [<60, 60–69, 70–79, and ≥80, respectively, unit (bpm)]. Analysis of variance (ANOVA) was used to compare the group differences. Principal sensitivity analysis was performed on participants who excluded self-reported diabetes and CVD, on this basis, participants diagnosed with atrial fibrillation were also excluded. Additional sensitivity analysis was performed on participants who were diagnosed with essential hypertension and heart failure. All statistical analyses were performed using R version 4.0.4 and *P*-value <0.05 was considered statistically significant (23).

#### 2.6.2. Two-sample MR

Two-sample MR is not prone to false-positive bias, which can occur in single-sample MR analyses. The summary statistics for the primary analysis were accomplished by the summary-level genome-wide analysis study (GWAS) of LV image-derived phenotypes in UK Biobank. The study using the LV metrics measured from CMR studies comprised 16,923 and 19,260 European UK Biobank participants with hearts of normal structure separately (24, 25). The secondary MR analysis used the results of GWAS meta-analysis including 47,309 heart failure cases and 930,014 controls (26). Palindromic SNPs were excluded and the remaining 20 SNPs among the summary statistics for RHR after clumping were utilized as genetic instruments. Inverse variance-weighted (IVW) method was applied to calculate the MR effect estimate of each LV metric, in addition, the robust penalized MR-Egger and the weighted median method were applied to reduce bias and evaluate validity of the instrumental variants (27, 28). The presence of cross-sectional polymorphism was assessed by the MR-Egger intercept test with a *P*-value <0.10 deemed to be evidence of polymorphic deviation (28). Leave-one-out analyses were conducted to assess the role of individual variants in driving the overall results. By calculating all these F statistics to avoid weak instrumental bias in MR analysis, the results were well above the commonly suggested threshold of F > 10 (29). All MR analyses were conducted using the R package (TwoSampleMR).

## 3. Results

### 3.1. Study population

Among a total of 502,469 participants in the UK Biobank, 26,892 participants had available CMR metrics excluded poor image quality data. After exclusion, our study included 20,062 individuals. Supplementary Figure S1 depicts a flowchart of the exclusion criteria and final study sample. The mean age of the participants was 54.9 ± 7.5 years and 47.5% were male with an average RHR of 68.9 ± 11.9 bpm. Baseline population characteristics stratified by RHR level are displayed in Table 1. Individuals with the highest RHR have higher BMI and blood pressure, meantime hypertension and diabetes were more prevalent in higher RHR groups compared to the lower.

**Table 1:** Characteristics of the study population stratified according to resting heart rate level.

	Mean (SD)				*P-*value for trend
Groups	<60 bpm	60–69 bpm	70–79 bpm	≥80 bpm	
	(*n* = 4,021)	(*n* = 6,716)	(*n* = 5,611)	(n = 3,837)	
Resting heart rate (bpm)	54.13 ± 4.27	64.57 ± 2.83	74.12 ± 2.81	87.81 ± 6.99	<0.001
Age (years)	54.44 ± 7.59	54.61 ± 7.42	55.07 ± 7.48	55.65 ± 7.31	<0.001
Male (%)	2,551 (63.4%)	3,263 (48.6%)	2,243 (40.0%)	1,540 (40.1%)	<0.001
Current drinker (%)	3,746 (93.2%)	6,248 (93.0%)	5,204 (92.7%)	3,454 (90.0%)	<0.001
Current smoker (%)	124 (3.1%)	244 (3.6%)	226 (4.0%)	163 (4.2%)	0.181
Physical Activity (%)					<0.001
Low	502 (12.5%)	1,055 (15.7%)	947 (16.9%)	694 (18.1%)	
Moderate	1,344 (33.4%)	2,329 (34.7%)	1,990 (35.5%)	1,375 (35.8%)	
High	1,592 (39.6%)	2,323 (34.6%)	1,743 (31.1%)	1,090 (28.4%)	
Body mass index (kg/m^2^)	25.71 ± 3.84	26.22 ± 4.12	26.71 ± 4.51	27.68 ± 4.97	<0.001
Body surface area (m^2^)	1.88 ± 0.19	1.86 ± 0.21	1.84 ± 0.21	1.86 ± 0.22	<0.001
Systolic blood pressure (mmHg)	140.31 ± 19.75	139.81 ± 19.50	140.05 ± 19.55	142.11 ± 19.70	<0.001
Diastolic blood pressure (mmHg)	76.44 ± 10.06	78.02 ± 10.26	79.17 ± 10.68	81.78 ± 10.77	<0.001
Self-reported history of					<0.001
Hypertension (%)	289 (7.2%)	608 (9.1%)	683 (12.2%)	627 (16.3%)	
Diabetes (%)	142 (3.5%)	266 (4.0%)	293 (5.2%)	342 (8.9%)	
Heart attack (%)	56 (1.4%)	59 (0.9%)	36 (0.6%)	27 (0.7%)	
Angina (%)	56 (1.4%)	50 (0.7%)	32 (0.6%)	24 (0.6%)	
Stroke (%)	25 (0.6%)	28 (0.4%)	25 (0.4%)	37 (1.0%)	
LV CMR parameters					
LVEDV (ml)	158.27 ± 34.21	142.34 ± 31.35	133.61 ± 29.57	128.18 ± 28.47	<0.001
LVESV (ml)	64.77 ± 18.82	57.19 ± 17.09	53.62 ± 16.31	52.43 ± 16.39	<0.001
LVEF (%)	59.21 ± 5.95	59.99 ± 5.98	59.99 ± 6.37	59.16 ± 7.02	<0.001
LV mass (g)	95.26 ± 21.39	88.87 ± 21.01	85.72 ± 19.92	85.71 ± 19.92	<0.001
WT (mm)	5.79 ± 0.73	5.69 ± 0.78	5.67 ± 0.76	5.61 ± 0.76	<0.001
Absolute GLS (%)	18.88 ± 2.62	18.66 ± 2.51	18.40 ± 2.58	17.98 ± 2.74	<0.001
MCF (%)	112.47 ± 18.02	111.25 ± 18.68	109.72 ± 19.06	105.18 ± 19.68	<0.001
LVGFI (%)	48.18 ± 6.24	48.16 ± 6.40	47.93 ± 6.58	46.75 ± 6.93	<0.001
RV CMR parameters
RVEDV (ml)	170.04 ± 38.68	152.15 ± 35.71	141.96 ± 33.58	136.07 ± 32.28	<0.001
RVESV (ml)	79.34 ± 22.06	70.09 ± 19.90	65.32 ± 18.99	63.35 ± 18.65	<0.001
RVEF (%)	53.55 ± 5.95	54.12 ± 5.96	54.16 ± 6.23	53.61 ± 6.75	<0.001

LV, left ventricular; RV, right ventricular; CMR, cardiac magnetic resonance; EDV, end-diastolic volume; ESV, end-systolic volume; SV, stroke volume; EF, ejection fraction; WT, wall thickness; GLS, global longitudinal strain; MCF, myocardial contraction fraction; LVGFI, left ventricular global function index.

### 3.2. Clinical associations

In the demographic table, people in the lower RHR group have larger ventricular structure. Due to this, we performed linear regressions to further study their cross-sectional correlation. Table 2 shows the average values of CMR metrics and the linear regression analysis results with sex differences shown in Supplementary Table S3.

**Table 2:** Associations between left and right ventricular magnetic resonance parameters and resting heart rate.

	Average value	Unadjusted			Adjusted		
	Mean ± SD	*β* (95% CI)	*P*	*P* for sex interaction	*β* (95% CI)	*P*	*P* for sex interaction
LV parameters
LVEDV (ml)	141.04 ± 32.77	−8.87 (−9.16 to −8.58)	<0.001	<0.001	−6.99 (−7.23 to −6.76)	<0.001	<0.001
LVESV (ml)	57.08 ± 17.74	−3.61 (−3.77 to −3.45)	<0.001	<0.001	−2.50 (−2.65 to −2.36)	<0.001	<0.001
LVSV (ml)	83.71 ± 19.39	−5.25 (−5.42 to −5.10)	<0.001	<0.001	−4.47 (−4.64 to −4.33)	<0.001	<0.001
LVEF (%)	59.68 ± 6.28	0.03 (−0.09 to 0.03)	0.27	<0.001	−0.28 (−0.34 to −0.22)	<0.001	<0.001
LV mass (g)	88.88 ± 20.94	−2.79 (−2.99 to −2.60)	<0.001	<0.001	−1.87 (−1.99 to −1.74)	<0.001	<0.001
LVWT (mm)	5.69 ± 0.77	−0.003 (−0.012 to 0.006)	0.54	<0.001	−0.021 (−0.032 to −0.010)	<0.001	<0.001
Absolute GLS (%)	18.48 ± 2.69	−0.29 (−0.32 to−0.26)	<0.001	<0.001	−0.37 (−0.42 to −0.31)	<0.001	<0.001
MCF (%)	109.73 ± 19.29	−3.04 (−3.22 to −2.87)	<0.001	<0.001	−2.94 (−3.26 to −2.61)	<0.001	0.002
LVGFI (%)	47.78 ± 6.70	−0.66 (−0.73 to −0.60)	<0.001	<0.001	−0.74 (−0.86 to −0.62)	<0.001	0.006
RV parameters
RVEDV (ml)	141.04 ± 32.77	−10.03 (−10.36 to −9.71)	<0.001	<0.001	−7.55 (7.80 to −7.30)	<0.001	<0.001
RVESV (ml)	57.08 ± 17.74	−4.72 (−4.91 to −4.54)	<0.001	<0.001	−3.26 (−3.42 to −3.11)	<0.001	<0.001
RVSV (ml)	83.71 ± 19.39	−5.31 (−5.49 to −5.13)	<0.001	<0.001	−4.28 (−4.45 to −4.12)	<0.001	<0.001
RVEF (%)	59.68 ± 6.28	0.02 (−0.04 to 0.08)	0.47	0.002	−0.18 (−0.25 to −0.12)	<0.001	<0.001

The model was adjusted for age, sex, ethnicity, socioeconomic status, alcohol consumption, smoking, physical activity, body mass index, hypertension, diabetes, and heart rate modifying medications.

The results are the effect size (95% confidence interval) for all left ventricular parameters per 10 beats per minute increase in resting heart rate.

LV, left ventricular; RV, right ventricular; EDV, end-diastolic volume; ESV, end-systolic volume; SV, stroke volume; EF, ejection fraction; WT, wall thickness; GLS, global longitudinal strain; MCF, myocardial contraction fraction; LVGFI, left ventricular global function index.

In the univariate unadjusted model, all CMR parameters except biventricular EF and LVWT were significantly correlated with RHR. After adjusting for covariates, higher RHR was still associated with smaller ventricular structure, lower ejection function and thinner wall thickness. Each 10 bpm increasing in RHR was correlated with a significant reduction in LVEDV of 6.99 ml [95% CI (−7.23 to −6.76); *P* < 0.001], LVEF of 0.28% [95% CI (−0.35 to −0.22); *P* < 0.001] and LVM of 1.87 g [95% CI (−1.99 to −1.74); *P* < 0.001]. Higher RHR was also linked to significantly unhealthy LV pathological remodeling, comprising lower MCF [*β* −2.94%, 95% CI (−3.26 to −2.61); *P* < 0.001]), LVGFI [*β* −0.74%, 95% CI (−0.86 to −0.62); *P* < 0.001] and GLS [*β* −0.37%, 95% CI (−0.42 to −0.31); *P* < 0.001].

**Figure 1:**
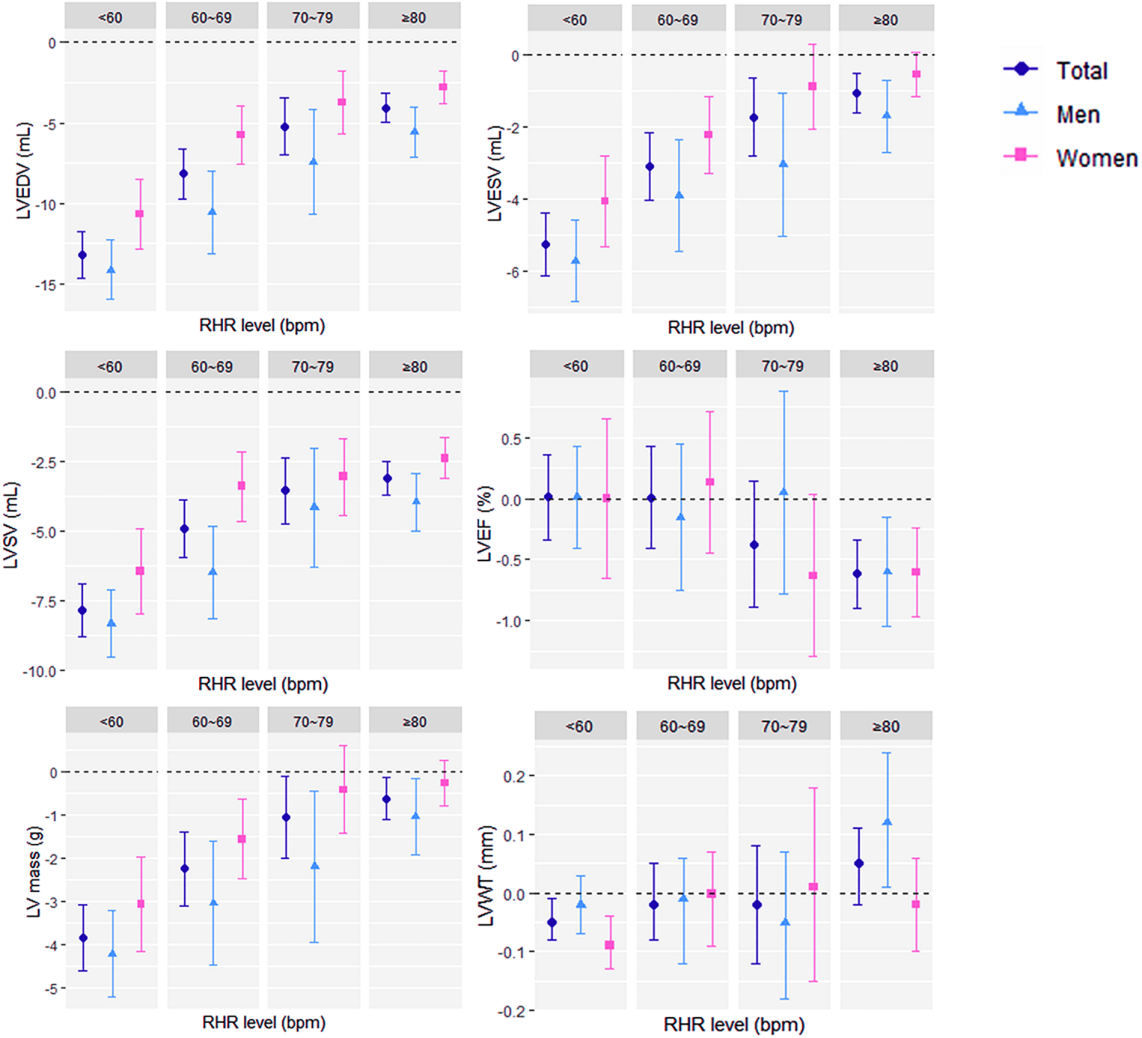
Associations (*β* with 95% confidence interval) of per 10 beats per minute increase in resting heart rate and left ventricular magnetic resonance parameters grouped by resting heart rate level (<60 bpm, 60–69 bpm, 70–79 bpm, and ≥80 bpm) in the fully adjusted model. RHR, resting heart rate; bpm, beats per minute; LV, left ventricular; CMR, cardiac magnetic resonance, EDV, end-diastolic volume; ESV, end-systolic volume; SV, stroke volume; EF, ejection fraction; WT, wall thickness.

All cardiac structural parameter reductions were greater in males (Supplementary Table S3), including ejection fraction. For example, LVEDV decreased by 8.86 ml in males [95% CI (−9.23 to −8.49); *P* < 0.001] for every 10 bpm increase in RHR but only about half of that in females [*β* 4.81 ml, 95% CI (−5.09 to −4.53); *P* < 0.001]. After grouping the population by RHR level, more detailed and clear results are presented in Figure 1. For ventricular end-diastolic and end-systolic volumes, the effect reduction was greatest at an RHR below 60 bpm. LVEF [*β* −0.62%, 95% CI (−0.90 to −0.34), *P* < 0.001] decreased significantly with each 10 bpm increase in RHR only in populations with RHR ≥ 80 bpm, and there were no sex differences in any group. We also found that in women, the associations between LVESV and LVM with RHR were not significant after the heart rate rose to 70 bpm or faster. For LVWT, there was a negative correlation in women with the lowest RHR and a positive correlation in men with the highest RHR. The differences between all groups were compared by ANOVA. The complete table of analysis results is shown in Supplementary Tables S4, S5.

Sensitivity analysis was first performed on individuals without atrial fibrillation, self-reported prevalent CVD and diabetes. Similar to the previous large population-based analysis, all cardiac parameters except RVEF were significantly negatively correlated with RHR, and all parameters had sex differences except LVEF. It is worth mentioning that LVWT has no correlation with RHR in healthy individuals. Next, in a population analysis of hospital-diagnosed essential hypertension, all cardiac morphological and functional parameters except LVWT significantly decreased as RHR increased, with no sex differences in biventricular ejection fraction. Heart failure patients with a hospital diagnosis were considered for the final analysis. The results demonstrated that all cardiac parameters were not significantly correlated to RHR in females. The findings for the subgroup of patients with heart failure are reported in Table 3, and the other results are displayed in Supplementary Tables S6, S7.

**Table 3:** Observational associations between CMR parameters and resting heart rate in the subgroup analysis of patients with heart failure.

	Heart failure patients (*n* = 223)		
	β (95% CI)	*P*	*P* for sex interaction
LV parameters
LVEDV (ml)
Total participants	−7.23 (−10.41 to −4.05)	<0.001	0.114
Female	−4.01 (−10.03 to 2.02)	0.19	
Male	−8.17 (−12.14 to −4.21)	<0.001	
LVESV (ml)
Total participants	−0.57 (−3.05 to 1.91)	0.65	0.625
Female	−1.65 (−6.19 to 2.89)	0.47	
Male	−0.81 (−3.97 to 2.35)	0.61	
LVSV (ml)
Total participants	−6.56 (−8.24 to −4.87)	<0.001	0.013
Female	−1.90 (−4.95 to 1.14)	0.22	
Male	−7.38 (−9.47 to −5.29)	<0.001	
LVEF (%)
Total participants	−1.84 (−2.77 to −0.91)	<0.001	0.367
Female	−0.02 (−1.92 to 1.89)	0.99	
Male	−2.03 (−3.15 to −0.90)	<0.001	
LV mass (g)
Total participants	−1.93 (−3.91 to 0.05)	0.06	0.201
Female	−1.15 (−4.68 to 2.39)	0.52	
Male	−1.95 (−4.55 to 0.66)	0.14	
LVWT (mm)
Total participants	0.08 (0.01 to0.16)	0.05	0.29
Female	0.02 (−0.14 to 0.19)	0.76	
Male	0.07 (−0.04 to 0.17)	0.22	
Absolute GLS (%)
Total participants	−1.06 (−1.48 to −0.63)	<0.001	0.31
Female	−1.44 (−2.73 to −0.75)	0.03	
Male	−0.91 (−1.38 to −0.43)	<0.001	
MCF (%)
Total participants	−5.37 (−7.48 to −3.25)	<0.001	0.40
Female	−3.63 (−8.28 to 1.02)	0.12	
Male	−4.94 (−7.57 to −2.31)	<0.001	
LVGFI (%)
Total participants	−1.88 (−2.80 to −0.96)	<0.001	0.28
Female	−1.67 (−3.94 to 0.60)	0.15	
Male	−1.57 (−2.69 to −0.45)	0.006	
RV parameters
RVEDV (ml)
Total participants	−7.40 (−10.39 to −4.41)	<0.001	0.018
Female	−3.95 (−8.69 to 0.80)	0.10	
Male	−8.60 (−12.45 to −4.74)	<0.001	
RVESV (ml)
Total participants	−0.67 (−2.71 to 1.38)	0.52	0.431
Female	−0.89 (−4.20 to 2.42)	0.59	
Male	−0.74 (−3.38 to 1.90)	0.58	
RVSV (ml)
Total participants	−6.73 (−8.51 to −4.96)	<0.001	0.002
Female	−3.06 (−5.94 to −0.18)	0.04	
Male	−7.85 (−10.17 to −5.54)	<0.001	
RVEF (%)
Total participants	−1.96 (−2.75 to −1.18)	<0.001	0.105
Female	−0.67 (−2.29 to 0.94)	0.99	
Male	−2.32 (−3.28 to −1.36)	<0.001	

The results are the effect size (95% confidence interval) for all parameters per 10 beats per minute increase in resting heart rate.

The model was adjusted for age, sex, ethnicity, socioeconomic status, alcohol consumption, smoking, physical activity, body mass index and systolic blood pressure.

LV, left ventricular; RV, left ventricular; CMR, cardiac magnetic resonance; EDV, end-diastolic volume; ESV, end-systolic volume; SV, stroke volume; EF, ejection fraction; WT, wall thickness; GLS, global longitudinal strain; MCF, myocardial contraction fraction; LVGFI, left ventricular global function index.

### 3.3. Two-sample MR results

**Figure 2:**
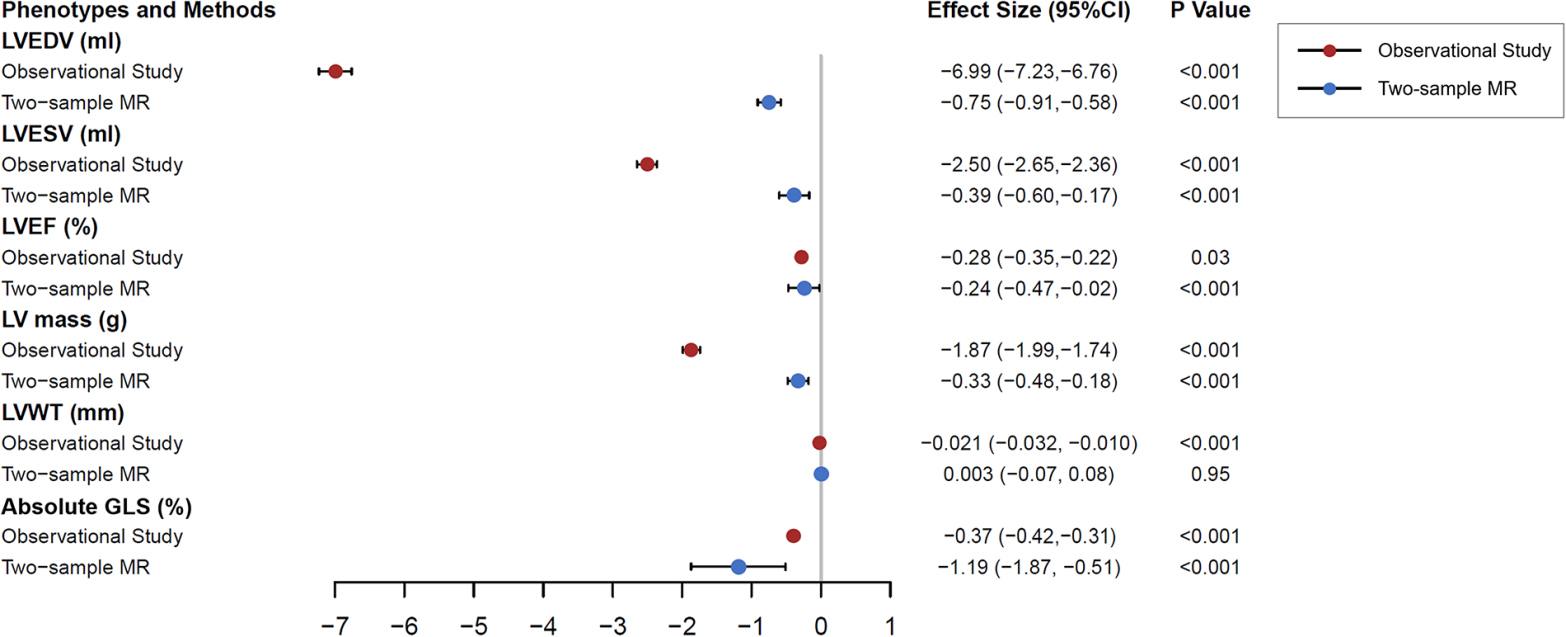
Clinical observation and two-sample Mendelian randomization analysis testing the effects of resting heart rate on left ventricular magnetic resonance structural parameters. The results are the causal estimates from inverse variance weighted approach and observational effects from multivariable linear regression model with 95% confidence interval, predicting per 10 beats per minute increase in resting heart rate on left ventricular parameters. The linear regression model was adjusted for age, sex, ethnicity, socioeconomic status, alcohol consumption, smoking, physical activity, body mass index, hypertension, diabetes, and heart rate modifying medications. MR, Mendelian randomization; LV, left ventricular; EDV, end-diastolic volume; ESV, end-systolic volume; EF, ejection fraction.

In the main analysis, the inverse variance weighting method combined with selected genetic variations gave a causal estimate of LV parameters as anticipated (Supplementary Table S8). All parameters except LVWT were statistically significant. The effect comparison between two-sample MR results of and the clinical observational study was shown in Figure 2. The causal estimation of LV structure by gene prediction was consistent with the direction of observational research, but the effect value was smaller. The genetically predicted RHR was not statistically significant in terms of causality with LVWT. As a sensitivity analysis, the weighted median MR method gave similar results to the main analysis, except for LVEF (*P* = 0.09). MR-egger analysis did not provide evidence of pleiotropy of RHR genetic variants on LV structure orientation, and further MR-egger intercept test did not show pleiotropy either. In the secondary analysis, all MR methods showed that there was no causation between RHR and heart failure. When we exclude SNPs that were closely related to possible major confounding factors by leave-one-out method, significant causal estimates were determined again. After this process, MR-egger pleiotropy test showed that directional pleiotropy was still not significant. Leave-one-out plots of genetic variants of RHR for causal estimates on each LV structural parameter are shown in Supplementary Figures S2A–F).

## 4. Discussion

### 4.1. Summary of results

This is the first study which involves both clinical and genetic analyses that systematically demonstrate associations between RHR and biventricular structure and function with sex differences using CMR assessment, independent of CVD and a wide range of clinical confounders.

Our clinical study suggests that higher RHR is linked with smaller ventricular structure (smaller EDV, ESV, and LV mass), poorer LV function (lower LVEF, GLS and LVGFI) and unhealthier pattern of LV remodeling (higher values of MCF). These trends are more pronounced among males. The impact of RHR on LVWT was negligible after grouping analysis. Additionally, MR analysis using inborn genetic instruments supported the causal effect of our clinical observational results. These observations are less affected by confounding or reverse causality, reflecting that RHR has an independent and broad impact on left ventricular remodeling.

### 4.2. Comparison with existing studies

The role of RHR in long-term development of CVD has been a topic of increasing research interest. It is well known that RHR is an independent predictor of all-cause and multiple cardiovascular mortality (30). These observations in this study add important evidence to the existing literature by demonstrating the correlation between RHR and ventricular remodeling parameters.

Several factors may influence the relationship of RHR to ventricular geometry. For the same cardiac output, it is a basic physiological principle that there is an inverse dependence between heart rate and stroke volume (7). The pacing-induced increase in heart rate, not accompanied by changes in tissue metabolic demand, causes a relative reduction in stroke volume mediated by a reduction in LVEDV but not with a corresponding change in ESV. RHR is considered a sensitive indicator of sympathetic and parasympathetic autoregulatory homeostasis (31, 32). A previous study of exercise testing among teenagers found that RHR as a separate genetic factor influenced overall cardiac vagal control (33). RHR mediated possible autonomic imbalance may be a cause of cardiac remodeling.

The concept of the heart rate vulnerable phase proposed in 2015 suggests that patients with heart failure at discharge with an RHR greater than 75 bpm will have increased the mortality and readmission rates (34). We found that high RHR weakened LV function including both LVEF, GLS and LVGFI, which might increase the risk and worsen the prognosis of heart failure. Zahra et al. also demonstrate LVGFI and LVEF were independent predictors of all-cause and CVD mortality, with larger effect sizes observed with LVGFI (35). However, it is worth mentioning that the decimal difference in EF (including GLS and LVGFI) is clinically insignificant. High RHR may lead to increased myocardial oxygen consumption, decreased myocardial perfusion, and finally decreased systolic function through the metabolic pathway of cardiomyocytes. Previous MR study showed that RHR had a negative causal effect on systolic blood pressure and a positive causal effect four times the absolute value above on diastole (36). This illustrates that the direct effect of RHR on cardiovascular diastole cannot be ignored. In addition, the decreased myocardial perfusion was due to shortened myocardial diastole, suggesting that increased heart rate may be directly related to diastolic heart failure (3). Higher RHR is closely related to lower MCF which represents hypertensive or pathological hypertrophy. As a preclinical stage of heart failure, hypertension is involved in inflammatory mechanisms and pathways similar to tachycardia-induced cardiomyopathy, suggesting that elevated RHR may play an important role in the progression of hypertension to heart failure (3, 37). However, our MR studies have shown that there is no causation between RHR and heart failure at the genetic level. Previous MR studies on heart failure have found that atrial fibrillation, body mass index and hypertension are risk factors independent of coronary heart disease (26). For RHR below 65 bpm in the AFGen cohort, there is an inverse causal association between genetically-determined RHR and incident atrial fibrillation (38). To sum up, RHR is regulated by possible mediating effects (atrial fibrillation, hypertension, etc.) at the genetic level, and ultimately affects the development and prognosis of heart failure at the clinical level.

The clinical associations between RHR and cardiac remodeling are particularly pronounced in men. As RHR levels increased above 70 bpm, the correlations remained in men, but not in women with LVESV and LVM. This is in line with a 2019 Japanese study showing that increased RHR was not significantly associated with the development of electrocardiographic left ventricular hypertrophy (ECG-LVH) in women, but was negatively correlated in men (39). However, in the subgroup analysis, the effect of RHR in the heart failure population was restricted to men. This may suggest that in the clinical treatment of CVD in men and women, the treatment of regulating heart rate probably benefit male patients more. The sex-specific differences suggest that heart rate-mediated cardiac morphological remodeling patterns differ in males and females, but ventricular function was not affected through some complex compensatory pathways, such as sex hormones. The cardiovascular protective effect of physiological testosterone remains controversial, but testosterone deficiency is common in men with dilated cardiomyopathy and heart failure. The mechanisms by which sex hormones regulate cardiac remodeling through genomic and nongenomic pathways remain to be elucidated.

We all know that RHR levels in endurance athletes are often below 60 bpm, or even below 40 bpm (40). Their hearts are stronger, with larger ventricular volumes and thicker LV walls, which may be related to nonpathological remodeling. Such hypertrophic remodeling reduces wall stress and maintains cardiac function and efficiency in response to increased load (37). Our study confirms that lower heart rate levels may lead to stronger myocardial hypertrophic remodeling, but it is more likely to be physiologic. A previous study showed that a lower heart rate over time is detrimental to electrocardiogram LV hypertrophy progression, which also suggests that RHR has two sides (39, 41). Although a higher RHR is associated with worse LV systolic function, in patients with heart failure and hypertension, heart rate-lowering drugs may accelerate the development of ventricular hypertrophy and disease progression.

### 4.3. Limitations

The present study has several limitations. First, cardiac activity and rates may be influenced by circadian rhythms, caffeine intake, body position and illness status such as infection or fever, which were not considered in the RHR collection conditions. Second, drugs such as ivabradine were not on the list of self-reported heart rate-modifying medications with blood serum data and sex hormone values not included in the adjustment factors. Third, the sample size of the subgroup analysis of heart failure patients was limited, so that the credibility and reproducibility of the results appear to require further validation. Finally, due to the insufficient effective SNPs of LV parameters’ GWAS summary after weak instrumental variable screening and clumping, reverse causal analysis cannot be carried out.

## 5. Conclusions

In this large population-based imaging study, we confirm higher RHR cause smaller ventricular chamber volume, poorer systolic function and unhealthy cardiac remodeling pattern. The differences between sex and disease subgroups complement the evidence providing validity for the underlying mechanisms of cardiac remodeling and help explore the potential scope or benefit of the intervention. However, ventricular hypertrophic remodeling caused by lower RHR poses new problems for heart rate control of clinical patients.

## Data Availability

The original contributions presented in the study are included in the article/**Supplementary Material**, further inquiries can be directed to the corresponding author/s.
