# Supplementary material for: Conventional and genetic associations between resting heart rate, cardiac morphology and function as assessed by magnetic resonance imaging: Insights from the UK biobank population study
Source: Front Cardiovasc Med. 2023 Mar 17;10:1110231. doi: 10.3389/fcvm.2023.1110231 (PMC10063878; doi:10.3389/fcvm.2023.1110231)
Supplement: Supplementary file 1 [file Table2.docx]

***Supplemental Material***

**Conventional and genetic associations between resting heart rate, cardiac morphology and function as assessed by magnetic resonance imaging: insights from the UK Biobank Population Study**

**Yao Ma^1^ †, Mengyao Qi^1^ †, Kexin Li^1^, Yuan Wang^1^, Fuxian Ren^2*^, Dengfeng Gao^1*^**

† These authors contributed equally to this work and share first authorship.

* Correspondence:

Corresponding author:

Dengfeng Gao: [gaomedic@mail.xjtu.edu.cn](mailto:gaomedic@mail.xjtu.edu.cn)

Fuxian Ren: [pyrfx518@163.com](mailto:pyrfx518@163.com)

1. Supplementary Figures and Tables
   1. Supplementary Figures

Supplementary Figure 1. Flowchart describing the sample selection.


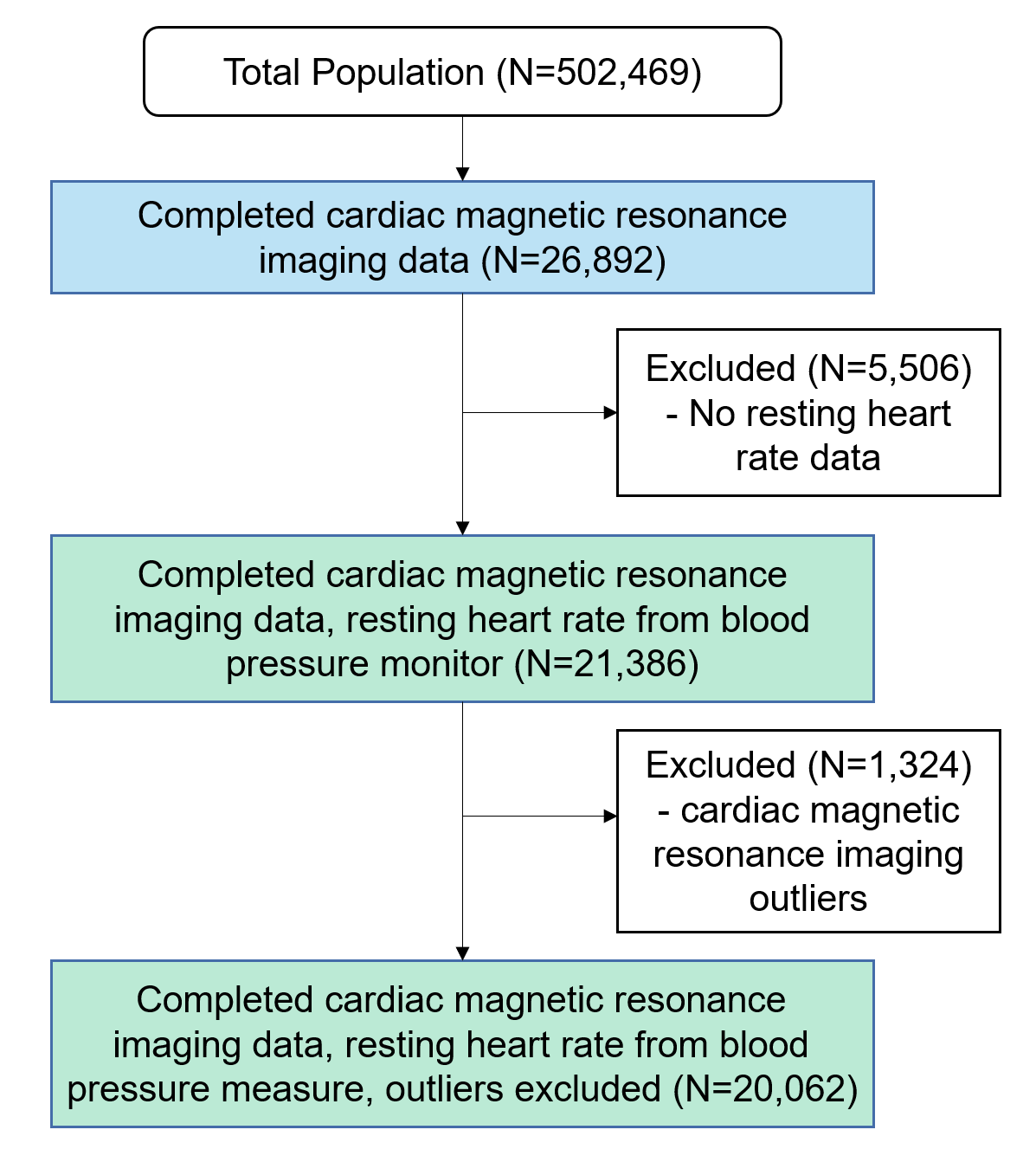


**Supplementary Figure 2.** Leave-one-out plots of genetic variants of resting heart rate for causal estimates on each left ventricular structural parameter.

**
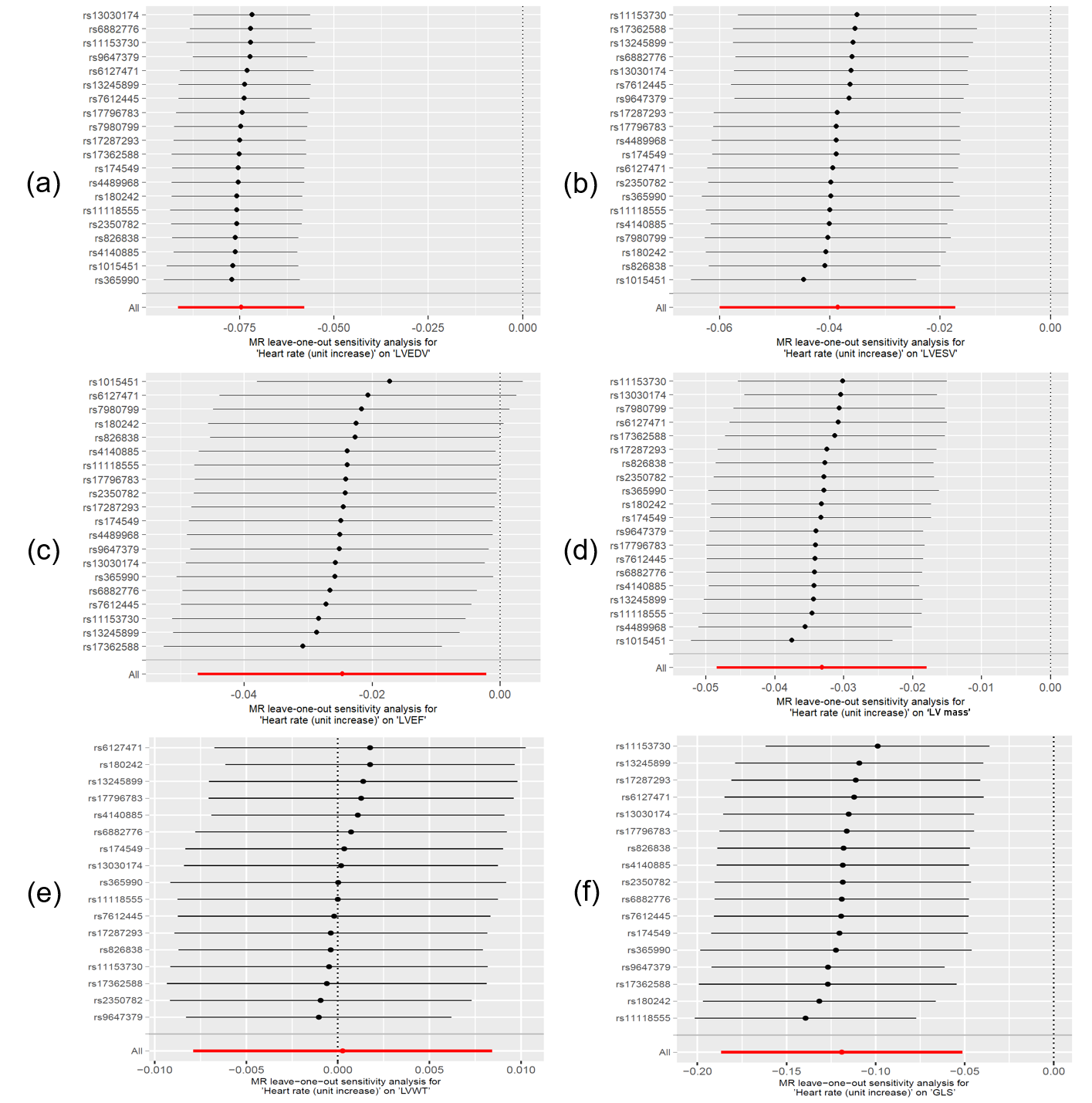
**

LV, left ventricular; EDV, end-diastolic volume; ESV, end-systolic volume; EF, ejection fraction; WT, wall thickness; GLS, global longitudinal strain.

- 1. Supplementary Tables

**Supplementary Table 1.** Genetic variants selected from a 2-stage meta-analysis of genome-wide association studies on resting heart rate.

| **SNP** | **Chromosome** | **Effect Allele** | **Non-effect allele** | **Beta** | **P Value** |
| --- | --- | --- | --- | --- | --- |
| rs11118555 | 1 | A | T | 0.612 | 4.00E-26 |
| rs13030174 | 2 | A | C | 0.3 | 1.00E-10 |
| rs17362588 | 2 | A | G | 0.736 | 4.00E-26 |
| rs4140885 | 2 | A | G | 0.217 | 5.00E-08 |
| rs13413635 | 2 | G | A | - | 3.00E-07 |
| rs7612445 | 3 | G | T | 0.358 | 2.00E-14 |
| rs9647379 | 3 | C | G | 0.206 | 1.00E-09 |
| rs2029213 | 3 | C | T | - | 7.00E-06 |
| rs10213084 | 4 | G | T | - | 4.00E-06 |
| rs6882776 | 5 | G | A | 0.301 | 2.00E-12 |
| rs7722600 | 5 | A | G | - | 3.00E-07 |
| rs1015451 | 6 | T | C | -0.713 | 1.00E-33 |
| rs11153730 | 6 | T | C | 0.381 | 8.00E-21 |
| rs236373 | 6 | C | T | - | 9.00E-07 |
| rs13245899 | 7 | G | A | 0.447 | 8.00E-27 |
| rs180242 | 7 | T | A | 0.316 | 7.00E-12 |
| rs2350782 | 7 | C | T | 0.505 | 1.00E-12 |
| rs174549 | 11 | A | G | 0.358 | 1.00E-22 |
| rs17287293 | 12 | A | G | 0.444 | 3.00E-20 |
| rs2067615 | 12 | A | T | 0.278 | 2.00E-09 |
| rs7980799 | 12 | A | C | 0.377 | 6.00E-24 |
| rs826838 | 12 | C | T | 0.234 | 4.00E-09 |
| rs11065706 | 12 | T | C | - | 6.00E-07 |
| rs17796783 | 14 | T | C | 0.334 | 3.00E-13 |
| rs365990 | 14 | G | A | 0.564 | 5.00E-45 |
| rs4489968 | 15 | T | G | 0.513 | 4.00E-20 |
| rs11645781 | 16 | A | G | - | 2.00E-07 |
| rs11083475 | 19 | G | A | - | 2.00E-06 |
| rs6127471 | 20 | C | T | 0.429 | 5.00E-29 |

**Supplementary Table 2.** Full list of rate-modifying medications identified from self to report and included in the fully adjusted model.

| **Oral beta to blocker preparations** |
| --- |
| Atenolol |
| bisoprolol |
| metoprolol |
| carvedilol |
| propranolol |
| inderal 10mg tablet |
| apsolol 10mg tablet |
| propanix 10mg tablet |
| sotalol |
| nebivolol |
| dorzolamide+timolol |
| atenolol+bendroflumethiazide |
| latanoprost+timolol |
| betaxolol |
| atenolol+bendrofluazide |
| nadolol |
| prindolol |
| Timolol |
| ethambutolol |
| pindolol |
| atenolol+chlortalidone |
| atenolol+nifedipine 50mg/20mg m/r capsule |
| atenolol+chlorthalidone |
| atenolol+co to amilozide |
| nadolol+bendroflumethiazide 40mg/5mg tablet |
| timolol maleate+bendroflumethiazide 10mg/2.5mg tablet |
| bisoprolol fumarate+hydrochlorothiazide 10mg/6.25mg tablet |
| celiprolol |
| labetalol |
| oxprenolol |
| acebutolol |
| propranolol hydrochloride+bendrofluazide 80mg/2.5mg capsule |
| sotalol hydrochloride+hydrochlorothiazide 80mg/12.5mg tablet |
| metoprolol tartrate+hydrochlorothiazide 100mg/12.5mg tablet |
| beta to blocker |
| tenormin 25 tablet |
| bedranol 10mg tablet |
| levobunolol |
| sotalol hydrochloride+hydrochlorothiazide 80mg/12.5mg tablet |
| propranolol hydrochloride+bendrofluazide 80mg/2.5mg capsule |
| carteolol |
| metoprolol tartrate+chlorthalidone 100mg/12.5mg tablet |
| half beta to prograne 80mg m/r capsule |
| beta to prograne 160mg m/r capsule |
| timolol maleate+co to amilozide 10mg/2.5mg/25mg tablet |
| cardinol 10mg tablet |
| **Non to dihydropyridine calcium channel blocker preparations** |
| cordilox 40mg tablet |
| adizem to xl plus m/r capsule |
| diltiazem |
| tildiem 60mg m/r tablet |
| verapamil |
| dilzem sr 60mg long acting m/r capsule |
| diltiazem hcl+hydrochlorothiazide 150mg/12.5mg m/r capsule |
| adizem to 60 m/r tablet |
| slozem 120mg m/r capsule |
| viazem xl 120mg m/r capsule |
| zemtard 120 xl m/r capsule |
| metazem 60mg m/r tablet |
| bi to carzem sr 60mg m/r capsule |
| adizem to xl plus m/r capsule |
| Angitil sr 90 m/r capsule |
| **Oral nitrate preparations (excluding GTN spray/sl)** |
| mycardol 30mg tablet |
| elantan 10 tablet |
| isosorbide mononitrate |
| ismn to isosorbide mononitrate |
| imdur 60mg durule |
| half to inderal la 80mg m/r capsule |
| isosorbide dinitrate |
| ismo to isosorbide mononitrate |
| isosorbide mononitrate product |
| xismox xl 60 m/r tablet |
| monomil xl 60mg m/r tablet |
| monomax sr 40 m/r capsule |
| isib 20mg tablet |
| ismo 10 tablet |
| isdn to isosorbide dinitrate |
| **Other rate modifying drugs** |
| amiodarone |
| digoxin |
| flecainide |
| digoxin product |
| medigoxin |

**Supplementary Table 3.** Observational associations between LV and RV CMR parameters and resting heart rate grouped by sex.

|  | **Unadjusted** | | | **Adjusted** | | |
| --- | --- | --- | --- | --- | --- | --- |
|  | **β (95%CI)** | **P** | **P for sex interaction** | **β (95%CI)** | **P** | **P for sex interaction** |
| LVEDV (mL) |  |  | <0.001 |  |  | <0.001 |
| Female | -4.59 (-4.89 to -4.29) | <0.001 |  | -4.81 (-5.09 to -4.53) | <0.001 |  |
| Male | -8.52 (-8.90 to -8.14) | <0.001 |  | -8.86 (-9.23 to -8.49) | <0.001 |  |
| LVESV (mL) |  |  | <0.001 |  |  | <0.001 |
| Female | -1.50 (-1.66 to -1.34) | <0.001 |  | -1.58 (-1.74 to -1.41) | <0.001 |  |
| Male | -3.23 (-3.44 to -3.01) | <0.001 |  | -3.30 (-3.53 to -3.07) | <0.001 |  |
| LVSV (mL) |  |  | <0.001 |  |  | <0.001 |
| Female | -3.09 (-3.29 to -2.89) | <0.001 |  | -3.24 (-3.44 to -3.04) | <0.001 |  |
| Male | -5.30 (-5.54 to -5.06) | <0.001 |  | -5.55 (-5.79 to -5.31) | <0.001 |  |
| LVEF (%) |  |  | <0.001 |  |  | <0.001 |
| Female | -0.25 (-0.34 to -0.17) | <0.001 |  | -0.27 (-0.37 to -0.18) | <0.001 |  |
| Male | -0.27 (-0.35 to -0.18) | <0.001 |  | -0.30 (-0.39 to -0.20) | <0.001 |  |
| LV mass (g) |  |  | <0.001 |  |  | <0.001 |
| Female | -0.32 (-0.48 to -0.16 | <0.001 |  | -1.07 (-1.21 to -0.93) | <0.001 |  |
| Male | -1.46 (-1.68 to -1.23) | <0.001 |  | -2.62 (-2.83 to -2.42) | <0.001 |  |
| LVWT (mm) |  |  | <0.001 |  |  | <0.001 |
| Female | 0.03 (0.02 to 0.04) | <0.001 |  | -0.04 (-0.05 to -0.02) | <0.001 |  |
| Male | 0.05 (0.04 to 0.06) | <0.001 |  | -0.02 (-0.03 to -0.01) | 0.048 |  |
| GLS (%) |  |  | <0.001 |  |  | <0.001 |
| Female | -0.28 (-0.33 to -0.24) | <0.001 |  | -0.31 (-0.38 to -0.23) | <0.001 |  |
| Male | -0.42 (-0.46 to -0.38) | <0.001 |  | -0.41 (-0.49 to -0.34) | <0.001 |  |
| MCF (%) |  |  | <0.001 |  |  | 0.002 |
| Female | -3.84 (-4.08 to -3.60) | <0.001 |  | -2.77 (-3.26 to -2.28) | <0.001 |  |
| Male | -4.01 (-4.23 to -3.79) | <0.001 |  | -3.04 (-3.47 to -2.60) | <0.001 |  |
| LVGFI (%) |  |  | <0.001 |  |  | 0.006 |
| Female | -0.95 (-1.04 to -0.87) | <0.001 |  | -0.64 (-0.82 to -0.45) | <0.001 |  |
| Male | -1.02 (-1.10 to -0.94) | <0.001 |  | -0.82 (-0.99 to -0.66) | <0.001 |  |
| RVEDV (mL) |  |  | <0.001 |  |  | <0.001 |
| Female | -5.23 (-5.54 to -4.91) | <0.001 |  | -5.48 (-5.77 to -5.18) | <0.001 |  |
| Male | -9.08 (-9.50 to -8.67) | <0.001 |  | -9.30 (-9.70 to -8.90) | <0.001 |  |
| RVESV (mL) |  |  | <0.001 |  |  | <0.001 |
| Female | -2.32 (-2.49 to -2.14) | <0.001 |  | -2.35 (-2.53 to -2.17) | <0.001 |  |
| Male | -3.97 (-4.22 to -3.73) | <0.001 |  | -4.05 (-4.30 to -3.80) | <0.001 |  |
| RVSV (mL) |  |  | <0.001 |  |  | <0.001 |
| Female | -3.09 (-3.29 to -2.89) | <0.001 |  | -3.13 (-3.33 to -2.93) | <0.001 |  |
| Male | -5.30 (-5.54 to -5.06) | <0.001 |  | -5.25 (-5.50 to -4.99) | <0.001 |  |
| RVEF (%) |  |  | 0.002 |  |  | <0.001 |
| Female | -0.25 (-0.34 to -0.17) | <0.001 |  | -0.10 (-0.19 to -0.01) | <0.001 |  |
| Male | -0.27 (-0.35 to -0.18) | <0.001 |  | -0.25 (-0.34 to -0.16) | <0.001 |  |

The model was adjusted for age, sex, ethnicity, socioeconomic status, alcohol consumption, smoking, physical activity, body mass index, hypertension, diabetes, and heart rate modifying medications.

The results are the effect size (95% confidence interval) for all left ventricular parameters per 10 beats per minute increase in resting heart rate.

LV, left ventricular; RV, right ventricular; EDV, end-diastolic volume; ESV, end-systolic volume; SV, stroke volume; EF, ejection fraction; WT, wall thickness; GLS, global longitudinal strain; MCF, myocardial contraction fraction; LVGFI, left ventricular global function index.

**Supplementary Table 4.** Observational associations between LV CMR parameters and resting heart rate grouped by resting heart rate level.

| **Resting Heart Rate Level (bpm)** | **<60** | | | **60~69** | | | **70~79** | | | **≥80** | | | **ANOVA P Value** |
| --- | --- | --- | --- | --- | --- | --- | --- | --- | --- | --- | --- | --- | --- |
|  | **β (95%CI)** | **P Value** | **P for sex interaction** | **β (95%CI)** | **P Value** | **P for sex interaction** | **β (95%CI)** | **P Value** | **P for sex interaction** | **β (95%CI)** | **P Value** | **P for sex interaction** |  |
| LVEDV (mL) |  |  |  |  |  |  |  |  |  |  |  |  |  |
| Total participants | -13.22 (-14.65 to -11.79) | <0.001 | <0.001 | -8.17 (-9.73 to -6.61) | <0.001 | <0.001 | -5.25 (-7.02 to -3.48) | <0.001 | 0.003 | -4.07 (-4.95 to -3.18) | <0.001 | <0.001 | <0.001 |
| Female | -10.69 (-12.84 to -8.53) | <0.001 |  | -5.77 (-7.57 to -3.96) | <0.001 |  | -3.72 (-5.69 to -1.76) | <0.001 |  | -2.78 (-3.79 to -1.77) | <0.001 |  | <0.001 |
| Male | -14.15 (-16.00 to -12.31) | <0.001 |  | -10.56 (-13.12 to -8.01) | <0.001 |  | -7.42 (-10.66 to -4.19) | <0.001 |  | -5.59 (-7.15 to -4.02) | <0.001 |  | <0.001 |
| LVESV (mL) |  |  |  |  |  |  |  |  |  |  |  |  |  |
| Total participants | -5.25 (-6.12 to -4.39) | <0.001 | <0.001 | -3.08 (-4.01 to -2.14) | <0.001 | <0.001 | -1.72 (-2.79 to -0.66) | <0.001 | 0.004 | -1.06 (-1.61 to -0.50) | <0.001 | <0.001 | <0.001 |
| Female | -4.06 (-5.31 to -2.81) | <0.001 |  | -2.22 (-3.27 to -1.16) | <0.001 |  | -0.89 (-2.05 to 0.28) | 0.14 |  | -0.55 (-1.16 to 0.07) | 0.08 |  | <0.001 |
| Male | -5.70 (-6.84 to -4.57) | <0.001 |  | -3.89 (-5.44 to -2.35) | <0.001 |  | -3.04 (-5.02 to -1.05) | <0.001 |  | -1.69 (-2.69 to -0.69) | <0.001 |  | <0.001 |
| LVSV (mL) |  |  |  |  |  |  |  |  |  |  |  |  |  |
| Total participants | -7.82 (-8.77 to -6.87) | <0.001 | <0.001 | -4.90 (-5.95 to -3.86) | <0.001 | <0.001 | -3.54 (-4.74 to -2.34) | <0.001 | 0.151 | -3.10 (-3.71 to -2.49) | <0.001 | 0.007 | <0.001 |
| Female | -6.44 (-7.96 to -4.91) | <0.001 |  | -3.38 (-4.64 to -2.12) | <0.001 |  | -3.03 (-4.42 to -1.65) | <0.001 |  | -2.36 (-3.09 to -1.64) | <0.001 |  | <0.001 |
| Male | -8.32 (-9.53 to -7.11) | <0.001 |  | -6.47 (-8.14 to -4.80) | <0.001 |  | -4.13 (-6.26 to -2.00) | <0.001 |  | -3.94 (-4.98 to -2.89) | <0.001 |  | <0.001 |
| LVEF (%) |  |  |  |  |  |  |  |  |  |  |  |  |  |
| Total participants | 0.01 (-0.34 to 0.36) | 0.95 | 0.135 | 0.01 (-0.41 to 0.43) | 0.97 | 0.673 | -0.37 (-0.89 to 0.14) | 0.16 | 0.078 | -0.62 (-0.90 to -0.34) | <0.001 | 0.156 | <0.001 |
| Female | 0.00 (-0.65 to 0.65) | 1.00 |  | 0.13 (-0.45 to 0.71) | 0.66 |  | -0.63 (-1.30 to 0.03) | 0.06 |  | -0.60 (-0.97 to -0.24) | <0.001 |  | <0.001 |
| Male | 0.01 (-0.40 to 0.43) | 0.95 |  | -0.15 (-0.75 to 0.45) | 0.62 |  | 0.05 (-0.78 to 0.88) | 0.91 |  | -0.60 (-1.05 to -0.16) | 0.01 |  | <0.001 |
| LV mass (g) |  |  |  |  |  |  |  |  |  |  |  |  |  |
| Total participants | -3.84 (-4.61 to -3.07) | <0.001 | <0.001 | -2.24 (-3.10 to -1.39) | <0.001 | <0.001 | -1.06 (-2.00 to -0.12) | <0.001 | <0.001 | -0.63 (-1.12 to -0.14) | 0.01 | <0.001 | <0.001 |
| Female | -3.06 (-4.17 to -1.96) | <0.001 |  | -1.56 (-2.48 to -0.64) | <0.001 |  | -0.42 (-1.43 to 0.59) | 0.41 |  | -0.26 (-0.79 to 0.26) | 0.32 |  | <0.001 |
| Male | -4.21 (-5.22 to -3.20) | <0.001 |  | -3.05 (-4.48 to -1.62) | <0.001 |  | -2.20 (-3.95 to -0.45) | 0.01 |  | -1.04 (-1.93 to -0.15) | 0.02 |  | <0.001 |
| LVWT (mm) |  |  |  |  |  |  |  |  |  |  |  |  |  |
| Total participants | -0.05 (-0.08 to -0.01) | 0.009 | <0.001 | -0.02 (-0.08 to 0.05) | 0.61 | 0.01 | -0.02 (-0.12 to 0.08) | 0.71 | 0.90 | 0.05 (-0.02 to 0.11) | 0.18 | 0.77 | <0.001 |
| Female | -0.09 (-0.13 to -0.04) | <0.001 |  | -0.001 (-0.09 to 0.07) | 0.80 |  | -0.05 (-0.18 to 0.07) | 0.41 |  | -0.02 (-0.10 to 0.06) | 0.60 |  | <0.001 |
| Male | -0.02 (-0.07 to 0.03) | 0.37 |  | -0.01 (-0.12 to 0.06) | 0.78 |  | 0.01 (-0.15 to 0.18) | 0.87 |  | 0.12 (0.01 to 0.24) | 0.04 |  | <0.001 |

The results are the effect size (95% confidence interval) for all left ventricular parameters per 10 beats per minute increase in resting heart rate.

The model was adjusted for age, sex, ethnicity, socioeconomic status, alcohol consumption, smoking, physical activity, body mass index, hypertension, diabetes, and heart rate modifying medications.

LV, left ventricular; CMR, cardiac magnetic resonance; EDV, end-diastolic volume; ESV, end-systolic volume; SV, stroke volume; EF, ejection fraction; WT, wall thickness.

**Supplementary Table 5.** Observational associations between RV CMR parameters and resting heart rate grouped by resting heart rate level.

| **Resting Heart Rate Level (bpm)** | **<60** | | | **60~69** | | | **70~79** | | | **≥80** | | | **ANOVA P Value** |
| --- | --- | --- | --- | --- | --- | --- | --- | --- | --- | --- | --- | --- | --- |
|  | **β (95%CI)** | **P Value** | **P for sex interaction** | **β (95%CI)** | **P Value** | **P for sex interaction** | **β (95%CI)** | **P Value** | **P for sex interaction** | **β (95%CI)** | **P Value** | **P for sex interaction** |  |
| RVEDV (mL) |  |  |  |  |  |  |  |  |  |  |  |  |  |
| Total participants | -13.52 (-15.07 to -11.96) | <0.001 | <0.001 | -8.71 (-10.38 to -7.03) | <0.001 | <0.001 | -5.08 (-6.97 to -3.19) | <0.001 | 0.006 | -5.13 (-6.07 to -4.18) | <0.001 | <0.001 | <0.001 |
| Female | -11.09 (-13.45 to -8.72) | <0.001 |  | -6.27 (-8.20 to -4.35) | <0.001 |  | -4.14 (-6.18 to -2.10) | <0.001 |  | -4.02 (-5.09 to -2.94) | <0.001 |  | <0.001 |
| Male | -14.40 (-16.39 to -12.41) | <0.001 |  | -11.08 (-13.83 to -8.32) | <0.001 |  | -6.49 (-10.00 to -2.97) | <0.001 |  | -6.46 (-8.13 to -4.78) | <0.001 |  | <0.001 |
| RVESV (mL) |  |  |  |  |  |  |  |  |  |  |  |  |  |
| Total participants | -6.94 (-7.91 to -5.96) | <0.001 | <0.001 | -4.08 (-5.10 to -3.05) | <0.001 | <0.001 | -1.63 (-2.79 to -0.47) | <0.001 | 0.025 | -2.13 (-2.71 to -1.54) | <0.001 | <0.001 | <0.001 |
| Female | -5.29 (-6.71 to -3.87) | <0.001 |  | -2.61 (-3.78 to -1.44) | <0.001 |  | -1.35 (-2.58 to -0.12) | 0.03 |  | -1.65 (-2.31 to -1.00) | <0.001 |  | <0.001 |
| Male | -7.62 (-8.90 to -6.35) | <0.001 |  | -5.52 (-7.22 to -3.82) | <0.001 |  | -2.18 (-4.36 to 0.00) | 0.05 |  | -2.70 (-3.76 to -1.65) | <0.001 |  | <0.001 |
| RVSV (mL) |  |  |  |  |  |  |  |  |  |  |  |  |  |
| Total participants | -6.58 (-7.58 to -5.57) | <0.001 | <0.001 | -4.63 (-5.73 to -3.53) | <0.001 | 0.001 | -3.45 (-4.69 to -2.21) | <0.001 | 0.037 | -3.00 (-3.63 to -2.37) | <0.001 | 0.005 | <0.001 |
| Female | -5.79 (-7.38 to -4.21) | <0.001 |  | -3.67 (-4.97 to -2.37) | <0.001 |  | -2.79 (-4.18 to -1.41) | <0.001 |  | -2.36 (-3.09 to -1.63) | <0.001 |  | <0.001 |
| Male | -6.78 (-8.05 to -5.50) | <0.001 |  | -5.56 (-7.35 to -3.76) | <0.001 |  | -4.31 (-6.55 to -2.06) | <0.001 |  | -3.75 (-4.84 to -2.67) | <0.001 |  | <0.001 |
| RVEF (%) |  |  |  |  |  |  |  |  |  |  |  |  |  |
| Total participants | 0.36 (0.00 to 0.72) | 0.05 | 0.093 | 0.06 (-0.36 to 0.49) | 0.78 | 0.225 | -0.57 (-1.08 to -0.06) | 0.03 | 0.648 | -0.18 (-0.45 to 0.10) | 0.21 | 0.151 | <0.001 |
| Female | 0.19 (-0.47 to 0.86) | 0.57 |  | -0.05 (-0.65 to 0.54) | 0.86 |  | -0.51 (-1.17 to 0.15) | 0.13 |  | -0.11 (-0.47 to 0.25) | 0.55 |  | <0.001 |
| Male | 0.46 (0.03 to 0.89) | 0.04 |  | 0.17 (-0.44 to 0.78) | 0.59 |  | -0.59 (-1.41 to 0.22) | 0.15 |  | -0.25 (-0.68 to 0.17) | 0.25 |  | <0.001 |

The results are the effect size (95% confidence interval) for all right ventricular parameters per 10 beats per minute increase in resting heart rate.

The model was adjusted for age, sex, ethnicity, socioeconomic status, alcohol consumption, smoking, physical activity, body mass index, hypertension, diabetes, and heart rate modifying medications.

RV, right ventricular; CMR, cardiac magnetic resonance; EDV, end-diastolic volume; ESV, end-systolic volume; SV, stroke volume; EF, ejection fraction.

**Supplementary Table 6.** Observational associations between CMR parameters and resting heart rate in the subgroup analysis of healthy individuals.

|  | **Self-reported healthy individuals (n=6547)** | | |
| --- | --- | --- | --- |
|  | **β (95%CI)** | **P Value** | **P for sex interaction** |
| **LV parameters** |  |  |  |
| LVEDV (mL) |  |  |  |
| Total participants | -7.25 (-7.74 to -6.76) | <0.001 | <0.001 |
| Female | -5.41 (-5.98 to -4.84) | <0.001 |  |
| Male | -9.28 (-10.11 to -8.46) | <0.001 |  |
| LVESV (mL) |  |  |  |
| Total participants | -2.62 (-2.91 to -2.33) | <0.001 | <0.001 |
| Female | -1.95 (-2.27 to -1.63) | <0.001 |  |
| Male | -3.38 (-3.87 to -2.88) | <0.001 |  |
| LVSV (mL) |  |  |  |
| Total participants | -4.65 (-4.98 to -4.31) | <0.001 | <0.001 |
| Female | -3.48 (-3.89 to -3.07) | <0.001 |  |
| Male | -5.92 (-6.47 to -5.38) | <0.001 |  |
| LVEF (%) |  |  |  |
| Total participants | -0.27 (-0.40 to -0.14) | <0.001 | 0.064 |
| Female | -0.20 (-0.38 to -0.01) | 0.03 |  |
| Male | -0.35 (-0.55 to -0.16) | <0.001 |  |
| LV mass (g) |  |  |  |
| Total participants | -1.88 (-2.13 to -1.62) | <0.001 | <0.001 |
| Female | -1.24 (-1.51 to -0.97) | <0.001 |  |
| Male | -2.69 (-3.13 to -2.25) | <0.001 |  |
| LVWT (mm) |  |  |  |
| Total participants | -0.007 (-0.017 to 0.002) | 0.25 | <0.001 |
| Female | -0.006 (-0.019 to 0.008) | 0.41 |  |
| Male | -0.01 (-0.03 to 0.003) | 0.12 |  |
| GLS (%) |  |  |  |
| Total participants | -0.41 (-0.46 to -0.35) | <0.001 | <0.001 |
| Female | -0.39 (-0.47 to -0.31) | <0.001 |  |
| Male | -0.42 (-0.50 to -0.34) | <0.001 |  |
| MCF (%) |  |  |  |
| Total participants | -3.38 (-3.77 to -2.98) | <0.001 | <0.001 |
| Female | -3.20 (-3.76 to -2.65) | <0.001 |  |
| Male | -3.41 (-3.96 to -2.85) | <0.001 |  |
| LVGFI (%) |  |  |  |
| Total participants | -0.86 (-1.00 to -0.71) | <0.001 | 0.003 |
| Female | -0.77 (-0.97 to -0.56) | <0.001 |  |
| Male | -0.92 (-0.11 to -0.72) | <0.001 |  |
| **RV parameters** |  |  |  |
| RVEDV (mL) |  |  |  |
| Total participants | -7.89 (-8.42 to -7.35) | <0.001 | <0.001 |
| Female | -5.89 (-6.50 to -5.29) | <0.001 |  |
| Male | -10.09 (-11.01 to -9.17) | <0.001 |  |
| RVESV (mL) |  |  |  |
| Total participants | -3.61 (-3.94 to -3.27) | <0.001 | <0.001 |
| Female | -2.63 (-2.99 to -2.27) | <0.001 |  |
| Male | -4.72 (-5.31 to -4.13) | <0.001 |  |
| RVSV (mL) |  |  |  |
| Total participants | -4.28 (-4.62 to -3.94) | <0.001 | <0.001 |
| Female | -3.26 (-3.67 to -2.86) | <0.001 |  |
| Male | -5.37 (-5.94 to -4.81) | <0.001 |  |
| RVEF (%) |  |  |  |
| Total participants | -0.10 (-0.23 to 0.04) | 0.16 | 0.009 |
| Female | -0.07 (-0.25 to 0.12) | 0.48 |  |
| Male | -0.12 (-0.32 to 0.08) | 0.25 |  |

The results are the effect size (95% confidence interval) for all left ventricular parameters per 10 beats per minute increase in resting heart rate.

The model was adjusted for age, sex, ethnicity, socioeconomic status, alcohol consumption, smoking, physical activity, body mass index and systolic blood pressure.

LV, left ventricular; RV, left ventricular; CMR, cardiac magnetic resonance; EDV, end-diastolic volume; ESV, end-systolic volume; SV, stroke volume; EF, ejection fraction; WT, wall thickness; GLS, global longitudinal strain; MCF, myocardial contraction fraction; LVGFI, left ventricular global function index.

Self-reported healthy individuals were defined as participants without self-reported prevalent cardiovascular disease and diabetes, meanwhile, participants diagnosed with atrial fibrillation were also excluded.

**Supplementary Table 7.** Observational associations between CMR parameters and resting heart rate in the subgroup analysis of patients with essential hypertension.

|  | **Essential hypertension (n=4351)** | | |
| --- | --- | --- | --- |
|  | **β (95%CI)** | **P Value** | **P for sex interaction** |
| **LV parameters** |  |  |  |
| LVEDV (mL) |  |  |  |
| Total participants | -7.33 (-7.89 to -6.76) | <0.001 | <0.001 |
| Female | -5.25 (-6.02 to -4.47) | <0.001 |  |
| Male | -8.38 (-9.15 to -7.61) | <0.001 |  |
| LVESV (mL) |  |  |  |
| Total participants | -2.36 (-2.72 to -2.00) | <0.001 | <0.001 |
| Female | -1.60 (-2.09 to -1.10) | <0.001 |  |
| Male | -2.74 (-3.23 to -2.25) | <0.001 |  |
| LVSV (mL) |  |  |  |
| Total participants | -5.00 (-5.37 to -4.63) | <0.001 | <0.001 |
| Female | -3.68 (-4.21 to -3.16) | <0.001 |  |
| Male | -5.67 (-6.17 to -5.17) | <0.001 |  |
| LVEF (%) |  |  |  |
| Total participants | -0.53 (-0.69 to -0.38) | <0.001 | 0.215 |
| Female | -0.41 (-0.67 to -0.15) | <0.001 |  |
| Male | -0.60 (-0.80 to -0.40) | <0.001 |  |
| LV mass (g) |  |  |  |
| Total participants | -2.05 (-2.37 to -1.72) | <0.001 | <0.001 |
| Female | -1.41 (-1.85 to -0.98) | <0.001 |  |
| Male | -2.36 (-2.80 to -1.92) | <0.001 |  |
| LVWT (mm) |  |  |  |
| Total participants | -0.001 (-0.008 to 0.005) | 0.66 | <0.001 |
| Female | -0.01 (-0.04 to 0.01) | 0.28 |  |
| Male | 0.01 (-0.01 to 0.03) | 0.31 |  |
| GLS (%) |  |  |  |
| Total participants | -0.39 (-0.42 to -0.35) | <0.001 | 0.33 |
| Female | -0.32 (-0.45 to -0.18) | <0.001 |  |
| Male | -0.44 (-0.53 to -0.34) | <0.001 |  |
| MCF (%) |  |  |  |
| Total participants | -2.31 (-2.52 to -2.10) | <0.001 | 0.002 |
| Female | -2.06 (-2.88 to -1.24) | <0.001 |  |
| Male | -2.59 (-3.14 to -2.05) | <0.001 |  |
| LVGFI (%) |  |  |  |
| Total participants | -0.53 (-0.61 to -0.45) | <0.001 | 0.004 |
| Female | -0.53 (-0.84 to -0.21) | 0.001 |  |
| Male | -0.64 (-0.86 to -0.42) | <0.001 |  |
| **RV parameters** |  |  |  |
| RVEDV (mL) |  |  |  |
| Total participants | -7.54 (-8.13 to -6.96) | <0.001 | <0.001 |
| Female | -5.34 (-6.12 to -4.56) | <0.001 |  |
| Male | -8.67 (-9.47 to -7.87) | <0.001 |  |
| RVESV (mL) |  |  |  |
| Total participants | -2.84 (-3.20 to -2.48) | <0.001 | <0.001 |
| Female | -1.92 (-2.39 to -1.44) | <0.001 |  |
| Male | -3.32 (-3.81 to -2.83) | <0.001 |  |
| RVSV (mL) |  |  |  |
| Total participants | -4.70 (-5.09 to -4.31) | <0.001 | <0.001 |
| Female | -3.42 (-3.97 to -2.88) | <0.001 |  |
| Male | -5.35 (-5.88 to -4.82) | <0.001 |  |
| RVEF (%) |  |  |  |
| Total participants | -0.45 (-0.60 to -0.29) | <0.001 | 0.541 |
| Female | -0.33 (-0.58 to -0.07) | <0.001 |  |
| Male | -0.50 (-0.69 to -0.31) | <0.001 |  |

The results are the effect size (95% confidence interval) for all left ventricular parameters per 10 beats per minute increase in resting heart rate.

The model was adjusted for age, sex, ethnicity, socioeconomic status, alcohol consumption, smoking, physical activity, body mass index and systolic blood pressure.

LV, left ventricular; RV, left ventricular; CMR, cardiac magnetic resonance; EDV, end-diastolic volume; ESV, end-systolic volume; SV, stroke volume; EF, ejection fraction; WT, wall thickness; GLS, global longitudinal strain; MCF, myocardial contraction fraction; LVGFI, left ventricular global function index.

Essential hypertension individuals were defined by hospital diagnostic data according to the International Classification of Diseases 10 (ICD-10).

**Supplementary Table 8.** Causal estimates from the two-sample MR analysis.

| **Phenotype and Methods** | **β (HR)** | **95%CI** | **P Value** |
| --- | --- | --- | --- |
| LVEDV (mL) |  |  |  |
| Inverse variance weighted | -0.75 | -0.91 to -0.58 | <0.001 |
| Weighted median | -0.63 | -0.83 to -0.42 | <0.001 |
| MR Egger | -0.38 | -0.89 to 0.12 | 0.15 |
| MR Egger intercept test |  |  | 0.15 |
| LVESV (mL) |  |  |  |
| Inverse variance weighted | -0.39 | -0.60 to -0.17 | <0.001 |
| Weighted median | -0.31 | -0.53 to -0.09 | 0.006 |
| MR Egger | -0.16 | -0.83 to 0.51 | 0.65 |
| MR Egger intercept test |  |  | 0.50 |
| LVEF (%) |  |  |  |
| Inverse variance weighted | -0.24 | -0.47 to -0.02 | 0.03 |
| Weighted median | -0.20 | -0.43 to -0.03 | 0.09 |
| MR Egger | -0.17 | -0.89 to 0.55 | 0.65 |
| MR Egger intercept test |  |  | 0.83 |
| LV mass (g) |  |  |  |
| Inverse variance weighted | -0.33 | -0.48 to -0.18 | <0.001 |
| Weighted median | -0.33 | -0.53 to -0.13 | 0.001 |
| MR Egger | -0.26 | -0.74 to 0.23 | 0.32 |
| MR Egger intercept test |  |  | 0.75 |
| LVWT (mm) |  |  |  |
| Inverse variance weighted | 0.003 | -0.07 to 0.08 | 0.95 |
| Weighted median | 0.02 | -0.08 to 0.12 | 0.66 |
| MR Egger | 0.02 | -0.24 to 0.29 | 0.86 |
| MR Egger intercept test |  |  | 0.87 |
| GLS (%) |  |  |  |
| Inverse variance weighted | -1.19 | -1.87 to -0.51 | <0.001 |
| Weighted median | -1.12 | -1.74 to -0.50 | <0.001 |
| MR Egger | -1.04 | -3.23 to -1.16 | 0.37 |
| MR Egger intercept test |  |  | 0.89 |
| Heart Failure |  |  |  |
| Inverse variance weighted | -0.05 | -0.21 to 0.11 | 0.53 |
| Weighted median | -0.10 | -0.27 to 0.06 | 0.21 |
| MR Egger | -0.26 | -0.81 to 0.28 | 0.36 |
| MR Egger intercept test |  |  | 0.43 |

The results are the causal effect size with 95% confidence interval for left ventricular parameters per 10 beats per minute increase in resting heart rate.

LV, left ventricular; EDV, end-diastolic volume; ESV, end-systolic volume; EF, ejection fraction; WT, wall thickness; GLS, global longitudinal strain.

Among the genetic instruments on LV parameters, rs2067615 was not included because it was palindromic SNP with intermediate allele frequency. Nine SNPs with null beta values were also excluded. The remaining 20 SNPs were used as valid genetic instruments. For the analysis of LVWT and GLS, three additional SNPs were also excluded due to missing required information.

Among the genetic instruments on heart failure, rs1015451, rs11118555, rs11645781, rs17083533, rs180242, rs2067615, rs4489968, rs7980799, and rs9647379 were not included because they were palindromic SNPs with intermediate allele frequency. Seven SNP with null beta values were also excluded. The remaining 14 SNPs were used as valid genetic instruments.
